# Perspective: The challenge of fraudulent participation in online nursing research

**DOI:** 10.1177/17449871261492946

**Published:** 2026-10-28

**Authors:** Jane Simanovski, Jody Ralph, Jamie Crawley, Harveer Mashiana, Siya Pandey, Edward Cruz

**Affiliations:** Assistant Professor, Faculty of Nursing, University of Windsor, Windsor, ON, Canada; Associate Professor, Faculty of Nursing, University of Windsor, Windsor, ON, Canada; Associate Professor, Faculty of Nursing, University of Windsor, Windsor, ON, Canada; BScN student, Faculty of Nursing, University of Windsor, Windsor, ON, Canada; BScN student, Faculty of Nursing, University of Windsor, Windsor, ON, Canada; Associate Professor, Faculty of Nursing, University of Windsor, Windsor, ON, Canada

## Introduction

Online research has revolutionised participant recruitment by allowing researchers to reach participants in geographically dispersed areas efficiently and cost-effectively (Comachio et al., 2025; Louis and Thompson, 2025). Social media have expanded researchers’ ability to reach populations that are difficult to access through traditional recruitment channels (Marston et al., 2026; Ménard et al., 2026). However, this same accessibility has introduced new vulnerabilities: the anonymity and scale of online platforms make it difficult to verify that respondents are credible and ‘real’ and increasing reliance on online recruitment methods has created challenges related to bots and fraudulent or imposter participants (Marston et al., 2026; Ménard et al., 2026).

Bots are automated software programs that perform online tasks without human intervention, whereas fraud involves deliberate attempts by individuals to misrepresent themselves for financial gain or other benefits (Louis and Thompson, 2025). Both can threaten the validity of research by introducing inaccurate or fabricated data (Towne and Polivka, 2026).

This growing prevalence of online recruitment has also increased the administrative burden associated with identifying, assessing and removing potentially fraudulent responses. The time required to review large numbers of suspicious entries highlights the importance of developing fraud detection and response procedures proactively, before recruitment begins (Marston et al., 2026; Ménard et al., 2026; Sharp et al., 2026). Researchers increasingly recognise that no single safeguard is sufficient on its own; maintaining data quality instead requires layered approach that begins during study design combined with ongoing human oversight applied across recruitment, data collection and cleaning (Comachio et al., 2025; Marston et al., 2026; Towne and Polivka, 2026).

Described are fraudulent survey activity during recruitment for an online study of lung transplant recipients’ informal caregivers, and the detection and mitigation strategies implemented to protect data quality and integrity. By situating our experience within the broader literature on online research vulnerabilities, we aim to contribute practical, study-tested guidance for research teams facing similar threats to data quality in web-based recruitment. This issue is particularly relevant to nursing research, where investigators increasingly rely on online recruitment strategies to engage geographically diverse, vulnerable and difficult-to-reach populations. Ensuring the authenticity of participant responses is therefore essential to maintaining the credibility, trustworthiness and applicability of nursing research findings.

## Study context

Following Research Ethics Board (REB) approval, our research team launched an online survey examining the experiences of informal caregivers supporting lung transplant recipients. Participation involved completing an approximately 20-minute online survey, and all participants who completed the survey were offered an electronic gift card as compensation. Given the cross-border nature of the study and the potential involvement of multiple lung transplant programmes, we recruited through the research associated social media and transplant-related social media sites. This strategy facilitated broader geographic reach and access to a more diverse caregiver population while avoiding reliance on any single transplant programme.

Initially, REB approved recruitment materials included a study flyer shared through the research specific social media account and/or community transplant organisations. The original flyer featured information about the study, inclusion criteria, a publicly accessible QR code linked directly to the survey, a single shared survey link that could be freely redistributed and a visually prominent electronic gift card compensation on the flyer to encourage participation.

Within 1 hour of the survey being posted on a research-oriented social media platform, 219 responses had been submitted. Although rapid recruitment is a recognised advantage of online research (Comachio et al., 2025; Crossen et al., 2026) the volume and speed of responses were unexpected given the relatively small and specialised population of interest: informal caregivers of lung transplant recipients. The unusually high response rate raised concerns regarding the authenticity and eligibility of participants and the potential presence of fraudulent submissions. As a result, the research team closed the survey to conduct a comprehensive assessment of data quality, review response patterns for indicators of bots and fraud and to evaluate the effectiveness of the recruitment strategy. Findings from this review informed subsequent modifications to the recruitment approach and implementation of additional steps to enhance data integrity.

## Indicators of fraudulent responses

Our experience revealed several vulnerabilities in the original study design that were consistent with those described in the broader literature (Comachio et al., 2025; Marston et al., 2026; Sharp et al., 2026). Firstly, although intended to facilitate legitimate participation, the publicly accessible QR code allowed entry into the survey without any pre-screening; the shared survey link had no mechanism to restrict or track redistribution, and the guaranteed compensation for completion, paired with visually prominent compensation language on the study flyer, may have inadvertently increased the study’s attractiveness to fraudulent respondents.

Across publications examining fraudulent participation in online research, researchers have identified a similar set of warning signs for distinguishing non-genuine responses, including unusually rapid response patterns, duplicate or shared IP addresses, geographic inconsistencies, repeated or inconsistent responses and indicators of limited content knowledge (Comachio et al., 2025; Crossen et al., 2026; Goodrich et al., 2023; Griffin et al., 2022). For example, our team identified 46 duplicate or triplicate IP addresses, one of the most cited indicators (Davies et al., 2024; Lei, 2024). Suspicious email patterns, such as multiple addresses with similar usernames, numbering patterns or other systematic similarities, were also considered potential indicators of fraudulent participation (Crossen et al., 2026; MacDonald et al., 2025).

Unrealistic completion times were a commonly cited indicator of fraudulent responses (Comachio et al., 2025; Matos et al., 2025). Consistent with this observation, our survey had an average completion time of 11 minutes, with many completed under 5 minutes, despite an anticipated completion time of 15–20 minutes. Rapid completion times were often considered alongside other indicators, such as AI-generated open-ended responses, when flagging potentially fraudulent entries (Matos et al., 2025). Inconsistent or implausible demographic responses, including conflicting answers to related questions elsewhere in the survey, were similarly used to identify fraudulent submissions (Lei, 2024; Webber-Ritchey et al., 2025). In our experience, inconsistencies in responses related to lung transplantation were also important indicators of potential fraud. These included conflicting diagnoses, discrepancies in reported age groups and inconsistencies in respondents’ stated relationships to transplant recipients. Finally, geographic mismatches, such as a respondent’s reported location conflicting with IP-based location data or eligibility criteria tied to a specific region, were used by several teams as an additional layer of verification (MacDonald et al., 2025; Marston et al., 2026). Similarly, our respondents were located in diverse geographic regions worldwide that were not expected based on our target population and recruitment strategy. Finally, although Qualtrics bot protection and Completely Automated Public Turing test to tell Computers and Humans Apart (CAPTCHA) features were enabled throughout data collection, 20 responses were still flagged by the platform as ‘potential bots’. Based on these assessments, the research team determined that the initial surveys’ responses were all potentially fraudulent or submitted by ineligible respondents. Consequently, the survey was closed, REB notified and the responses excluded from analysis.

### Mitigation strategies

Our team amended the study protocol by implementing a multi-layered mitigation strategy that included: (1) modifications to the study flyer and recruitment materials; (2) implementation of an eligibility screening process; (3) enhanced controls over survey access; and (4) ongoing data monitoring to identify potentially fraudulent responses.

Recruitment materials were revised to reduce the emphasis on financial incentives. The guaranteed gift card was replaced with a raffle-based incentive, and visual references to monetary rewards were removed from recruitment advertisements. This modification was informed by methodological literature suggesting that incentive structures can influence the attractiveness of online surveys to fraudulent or bot-generated respondents (Goodrich et al., 2023; Griffin et al., 2022). Furthermore, following the revised REB approval, we implemented an eligibility pre-screening survey as an additional measure to mitigate fraudulent survey activity. Prospective participants were asked a short series of questions requiring knowledge of the lung transplantation and associated caregiving experience, including caregiving responsibilities, post-transplant milestones and follow-up care procedures. Using Qualtrics survey logic and branching functions, respondents were required to successfully complete the pre-screening survey before they could progress to the final screen and provide an email address to receive the link to the full survey. This approach enabled manual verification of eligibility while creating an additional barrier to automated bots and fraudulent respondents. Eligible participants were then sent individualised Qualtrics survey links only after their pre-screening responses had been reviewed. Each link was associated with the email address used by the eligible participant during pre-screening, thereby restricting survey access to the intended participant and reducing the opportunity to forward or share the link or access the full survey through multiple email addresses.

Throughout recruitment, the responses from both eligibility and actual surveys were continuously monitored for indicators of potential fraudulent participation, including unusually rapid completion times, repeated response patterns and inconsistent demographic information. Technical safeguards, including CAPTCHA verification to help distinguish human participants from automated programmes, IP screening to identify potentially duplicate submissions or unusual geographic patterns and attention-check questions to assess participant engagement, were incorporated to strengthen fraud detection. Responses that met the predefined criteria for potential fraudulent participation were excluded from the analytic dataset.

### Lessons learned

Our experience demonstrated that effective fraud prevention requires a layered approach that begins during study design rather than after fraudulent responses are detected. Our experience is consistent with recent literature demonstrating that fraudulent participation can be identified through patterns in recruitment volume, timing, email characteristics, IP addresses and the quality or authenticity of participant responses (Crossen et al., 2026). Although CAPTCHA, IP screening and attention checks provided useful indicators of potentially fraudulent activity, none could independently verify respondent legitimacy. Accordingly, these measures were used as part of a broader fraud prevention strategy that included eligibility screening, controlled survey access, ongoing data monitoring and manual review of suspicious responses (Comachio et al., 2025).

Caregiver-specific pre-screening questions were particularly valuable because they required experience-based knowledge of the lung transplant caregiving role that would be difficult for fraudulent respondents to consistently fabricate. These questions provided an additional layer of verification beyond basic eligibility criteria and are consistent with current literature emphasising the value of assessing participant content expertise and the authenticity of participant experiences when evaluating potentially fraudulent participation in online research (Comachio et al., 2025; Crossen et al., 2026). Importantly, content expertise was considered as one component of the broader fraud assessment process rather than as a standalone criterion for determining fraudulent participation. In addition, reducing the emphasis on financial incentives by replacing a guaranteed gift card with a raffle-based incentive may have reduced the study’s appeal to imposter participants.

Although recruitment remains ongoing, fewer than half of respondents who completed the pre-screening survey progressed to the full survey thus far, as a considerable proportion were screened out because of concerns regarding eligibility or indicators of potentially fraudulent participation. It also highlights the need to allocate sufficient resources for ongoing recruitment monitoring, manual review and systematic data cleaning while implementing multiple complementary safeguards to protect data integrity in online research.

## Conclusion

The rapid emergence of fraudulent responses in our study demonstrated how vulnerable online research can be various forms of deceptive participation. Beyond the methodological challenges, responding to fraudulent activity imposes a considerable administrative and emotional burden on the research team.

Our experience reinforced the need to anticipate fraudulent participation during the study design phase rather than responding only after concerns arise. Integrating preventative safeguards can reduce the risk of compromised data, lessen the burden associated with extensive data cleaning and improve the efficiency of recruitment efforts. Nursing researchers increasingly rely on web-based recruitment to engage hard-to-reach populations. Fraud prevention should be regarded as an essential component of study design. Research ethics boards, funding agencies and investigators may need to view participant verification processes as fundamental to protecting research integrity. Drawing on our experience, Figure 1 presents a Checklist for preventing fraudulent participation in online nursing research that may assist researchers in designing and conducting rigorous web-based survey studies.

**Figure 1.**
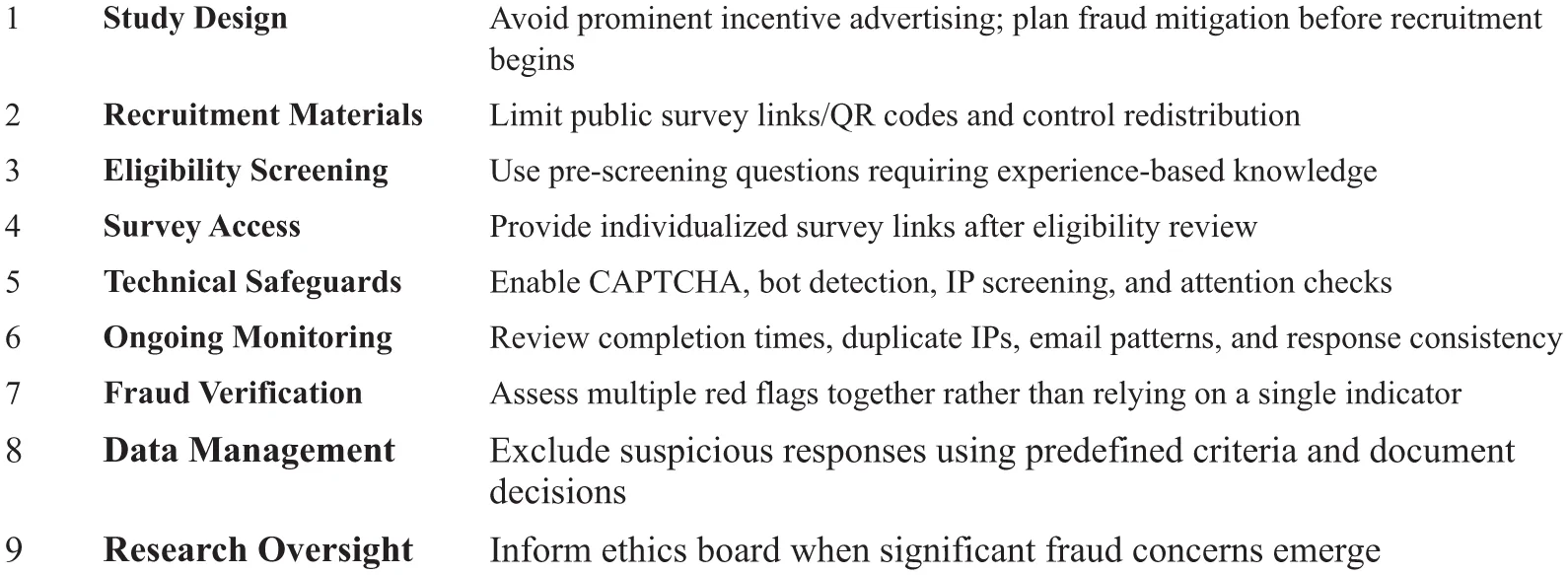
Checklist for preventing fraudulent participation in online nursing research.

**Key points for policy, practice and research**

- Online studies are vulnerable to bots and fraudulent respondents.
- Multiple indicators can help identify suspicious survey activity early.
- Layered fraud mitigation strategies improve participant verification.
- Proactive prevention measures are essential to protect data quality and integrity in nursing research.
